# Beyond the Surgical Bill: Pharmacoeconomics and Real-World Utilization Across the Knee Osteoarthritis Care Pathway—A Critical Narrative Review

**DOI:** 10.3390/healthcare14142066

**Published:** 2026-07-09

**Authors:** Furkan Yapıcı

**Affiliations:** Department of Orthopedics and Traumatology, Erzincan Binali Yıldırım University, 24100 Erzincan, Turkey; furkanyapici@hotmail.com

**Keywords:** knee osteoarthritis, pharmacoeconomics, pharmacoepidemiology, real-world evidence, drug utilization, NSAIDs, opioids, intra-articular injections, healthcare resource utilization, arthroplasty

## Abstract

**Background:** Knee osteoarthritis (KOA) is often framed as degenerative knee pain, yet behaves as a decades-long care pathway in which medication, injections, comorbidity, productivity loss, and surgery accumulate into a major economic footprint. This critical narrative review synthesizes pharmacoeconomic and pharmacoepidemiologic evidence across that pathway. **Methods:** Structured source identification was conducted in PubMed, Web of Science, Scopus, and Google Scholar for publications from 2000 to 2026, with citation tracking. Sources were appraised against predefined critical-interpretation domains and mapped narratively rather than pooled; no meta-analysis was performed. **Results:** Global Burden of Disease 2019 estimates approximately 364.6 million prevalent KOA cases worldwide. Reported evidence indicates that KOA spending is highly concentrated: in a large U.S. claims analysis, knee arthroplasty was performed in approximately 8.8% of patients yet accounted for 61.5% of KOA-related costs, whereas hyaluronic acid represented 3.0% of overall costs; the remaining pathway burden was distributed across years of outpatient care, analgesics, injections, and other nonsurgical utilization. Medication and injection findings were stage- and phenotype-dependent, and observational studies associated opioid exposure with higher fall risk, healthcare utilization, and cost. Intra-articular hyaluronic acid was repeatedly associated with longer time to arthroplasty, interpreted here as an association limited by confounding and immortal-time bias, not a causal effect; platelet-rich plasma value remained price- and durability-sensitive. **Conclusions:** KOA economics resembles an iceberg—arthroplasty is the visible peak, while the submerged mass is years of pathway-level care. Value-based policy should measure the full pathway, not the surgical episode, using linked claims, registries, patient-reported outcomes, and productivity data.

## 1. Introduction

Knee osteoarthritis (KOA) is often introduced as “degenerative knee pain,” yet its clinical course and economic footprint behave like those of a long-horizon chronic disease with stage-dependent escalation of resource use. A core interpretive challenge is case definition. Radiographic KOA (structural change) and symptomatic KOA (structural change plus clinically meaningful symptoms) differ in disability and health-related quality of life, and therefore in the utilities and quality-adjusted life-years (QALYs) used in cost–utility analyses; the same definitional choice can materially change both economic attribution and incremental cost-effectiveness ratios (ICERs) [1]. Because these utilities are typically derived from patient-reported outcome measures (PROMs), the validity, reliability, and responsiveness of the instruments used to capture knee symptoms and function also bear effect on the resulting economic estimates [2].

From a pharmacoepidemiologic perspective, KOA is not only a disorder of structural joint degeneration but also a long-duration medication, injection, device, procedure, and claims-data problem. Over the care pathway, patients may cycle through topical and oral nonsteroidal anti-inflammatory drugs (NSAIDs), acetaminophen, duloxetine, opioids, intra-articular corticosteroids, intra-articular hyaluronic acid (IA-HA), platelet-rich plasma (PRP), bracing, physical therapy, procedural interventions, arthroplasty, and revision care. Each exposure generates distinct utilization signals, safety concerns, and payer costs, so KOA pharmacoeconomics is best interpreted at the pathway level rather than as a set of isolated treatment episodes. This matters because major guidelines broadly agree on core non-pharmacologic care and cautious NSAID use but diverge on acetaminophen, symptomatic slow-acting drugs, IA-HA, PRP, opioids, and some procedural interventions [3,4,5,6,7,8]—divergences that become economically consequential when cost-effectiveness is judged against conventional willingness-to-pay (WTP) thresholds.

The scale of KOA is global and rising, and its economic weight is amplified by chronicity: because symptomatic disease is typically lived with for decades, time horizon and discounting become decisive for any intervention intended to delay surgery, and KOA frequently behaves as a high-utilization phenotype intertwined with comorbidity and all-cause spending rather than as a narrow diagnostic cost silo [9,10,11]. Critically, spending is not evenly distributed along the pathway. Arthroplasty is the most visible and concentrated cost, yet it is the tip of a less-visible, longitudinal burden composed of years of outpatient care, analgesic and injection cycling, healthcare resource utilization, and productivity loss—an “economic iceberg” in which procedure-level debates (for example, high tibial osteotomy versus unicompartmental arthroplasty) sit inside a broader cost architecture. Much economic evaluation has nonetheless focused on the surgical episode in isolation, and conclusions diverge across studies because they differ in KOA definition, phenotype mix, time horizon, analytic perspective (payer versus societal), costing basis, and accounting of downstream events such as complications, reoperations, and revisions [1,11,12].

For clinicians and payers, the central question is therefore not whether KOA is costly but where along the care pathway avoidable cost, harm, and delayed value arise—because the decisions that shape cumulative cost are clinical before they are economic. This question is increasingly consequential as health systems adopt value-based and bundled-payment models that hold care accountable across episodes rather than at the level of single procedures, making the full-pathway view a practical as well as a conceptual concern. Accordingly, this critical narrative review synthesizes pharmacoeconomic and real-world utilization evidence across the KOA care pathway. Its objective is to identify how guideline recommendations, medication exposure, analgesic persistence, NSAID contraindications, opioid use, intra-articular injections, biologic and device-based interventions, healthcare resource utilization, and arthroplasty-related downstream events shape costs and value. Rather than treating KOA economics as a series of independent cost estimates, the review interprets the evidence as a longitudinal care pathway in which early treatment choices, coverage, comorbidity, pain severity, and the timing of surgery alter cumulative costs, QALYs, and policy conclusions.

## 2. Methods

### 2.1. Review Type and Objective

This article was designed as a critical narrative review of pharmacoeconomic and real-world utilization evidence in knee osteoarthritis (KOA), rather than as a systematic review, scoping review, or meta-analysis. The objective was to synthesize decision-relevant evidence on treatment utilization, cost drivers, cost-effectiveness, and downstream economic consequences across the KOA care pathway, integrating claims and registry studies, pharmacoeconomic evaluations, guideline documents, drug- and injection-utilization studies, and arthroplasty-related cost analyses.

Consistent with this design, a registered protocol, duplicate independent screening, duplicate data extraction, formal risk-of-bias scoring, and quantitative pooling were not undertaken. Methodological transparency was instead strengthened through a structured source-identification strategy, predefined source-prioritization criteria, evidence-family classification, population-specific labeling, source charting, and an explicit critical-interpretation framework (Section 2.4). The aim was a critical, decision-oriented synthesis of heterogeneous evidence rather than exhaustive ascertainment of all eligible studies or pooled effect estimation; accordingly, no meta-analysis was performed, and the manuscript should not be interpreted as a systematic review.

### 2.2. Scope of the Review

The review focused on adult KOA and on evidence relevant to pharmacoeconomics, pharmacoepidemiology, real-world utilization, and pathway-level value. Symptomatic and/or radiographic KOA were included when explicitly reported, together with selected knee/hip osteoarthritis (OA) or broader OA studies when these provided decision-relevant context for medication exposure, healthcare resource utilization (HCRU), opioid use, NSAID contraindications, or economic burden. To keep this distinction visible, every source is labeled by population scope—KOA-specific, knee/hip OA, or OA-wide (Supplementary Table S1)—and non-KOA-specific evidence is explicitly flagged as supportive rather than KOA-specific at first mention in the Results.

The evidence was organized across the KOA care pathway: guideline-recommended care; conservative management; pharmacologic exposure; NSAID and opioid utilization; intra-articular corticosteroid, hyaluronic acid (IA-HA), and platelet-rich plasma (PRP) use; HCRU; pre-arthroplasty spending; arthroplasty and joint-preserving surgery; postoperative utilization; bundled payments; and methodological challenges in real-world economic evidence.

### 2.3. Source Identification

PubMed, Web of Science, Scopus, and Google Scholar were searched, with supplementary citation tracking. PubMed served as the primary biomedical database; Web of Science and Scopus supported citation tracking and cross-disciplinary identification of economic and health-services research. Google Scholar was used as a supplementary, relevance-ranked source rather than as a primary database: the first 100 results of the main combined search were screened, because relevance ranking declines substantially beyond the early results in broad narrative searches, and the aim was to capture additional economic models, institutional full texts, and recently indexed sources rather than to retrieve all records exhaustively. Publications issued between 1 January 2000 and 30 April 2026 were considered, with searches conducted between 1 April and 30 April 2026. Full search concepts and representative database search strings are provided in Supplementary Materials.

No geographic restrictions were applied. Because cost estimates are highly sensitive to reimbursement structure, currency, price year, payer system, and site of care, cross-country results were interpreted qualitatively rather than pooled. Cost values are deliberately retained in the original currency and price year of each source, without conversion or inflation adjustment, to avoid introducing spurious cross-country comparability; currency, cost year, country, analytic perspective, and costing basis are reported alongside economic estimates wherever available.

### 2.4. Operationalization of the Critical Narrative Approach

To make the critical component explicit and reproducible, each source was appraised and interpreted against a predefined set of domains rather than by formal risk-of-bias scoring: (i) population specificity (KOA-specific vs. knee/hip OA vs. OA-wide); (ii) disease definition (symptomatic, radiographic, clinical, or claims-coded); (iii) cost attribution (all-cause vs. OA-related vs. KOA-attributable vs. treatment-specific); (iv) exposure definition (route, dose, persistence, switching, number of injection courses, and timing relative to outcomes); (v) comparator choice; (vi) analytic perspective (payer, healthcare-system, patient, or societal); (vii) time horizon, discounting, and completeness of downstream-event capture; (viii) cost year, currency, and costing basis; (ix) confounding by indication and channeling bias; (x) disease-stage misclassification; (xi) immortal-time and time-dependent exposure bias, particularly in delay-to-arthroplasty analyses; (xii) transferability across health systems; and (xiii) funding source, declared conflicts of interest, and commercial evidence density.

Evidentiary weight was judged by design strength, directness to the decision question, population specificity, and completeness of economic reporting. Primary claims, registry, cohort, trial-based, and model-based studies were preferred for numerical statements, whereas prior systematic and narrative reviews were used to contextualize evidence families rather than as primary support for quantitative conclusions. Conflicting findings were weighed by considering population match, study design, analytic perspective, time horizon, and completeness of downstream-event capture, rather than by vote-counting; apparently discordant results were, wherever possible, interpreted as consequences of these methodological differences. Commercially sensitive evidence—most notably for IA-HA and PRP—was appraised with explicit attention to funding source and conflicts of interest. Funding source, declared conflicts of interest, and commercial relevance were recorded in Supplementary Table S1 where reported or directly relevant to interpretation, with particular attention paid to commercially dense intervention areas such as IA-HA and PRP. Funding or commercial relevance was not used as an exclusion criterion but as an interpretive caution, and evidence volume was not treated as evidence strength or as proof of pathway priority, particularly for commercially dense intervention areas such as IA-HA and PRP. Throughout, findings from claims and registry data are presented as real-world associations unless the underlying design supports stronger causal inference.

### 2.5. Source Transparency and Evidence Mapping

**Figure 1:**
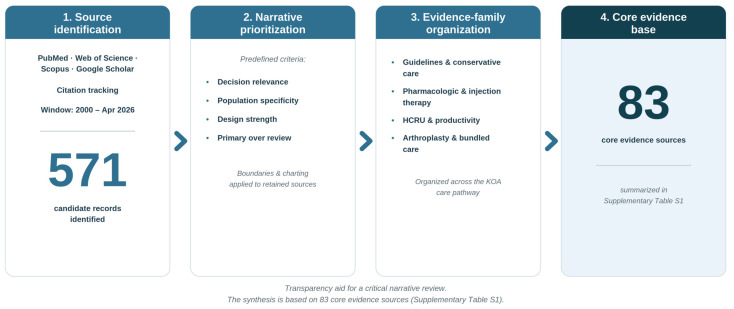
Source-transparency map of the critical narrative review. The figure summarizes source identification, narrative prioritization, evidence-family organization, and the final core evidence base used for the synthesis. The synthesis is based on 83 core evidence sources, summarized in Supplementary Table S1. This figure is provided as a transparency aid. It is not a formal systematic-review flowchart or PRISMA-style flow diagram.

The source-identification process is summarized as a transparency map (Figure 1) and a descriptive source-identification table (Supplementary Table S2). Figure 1 is a source-transparency map and should not be interpreted as a formal systematic-review flowchart or PRISMA-style flow diagram. These are transparency aids that summarize how sources were identified and organized for this critical narrative review. The synthesis is based on 83 core evidence sources, summarized in Supplementary Table S1. Additional references are cited only for contextual purposes and are not counted as core evidence sources.

Supplementary Table S1 summarizes the core evidence sources used for the synthesis—by population label, design, pathway stage, perspective, currency and cost year, economic outcome, funding/conflict status and commercial relevance, and major limitation—and Supplementary Table S2 provides the descriptive source-identification summary. Reasons for non-prioritizing full-text reports not carried into the core evidence set are summarized in Supplementary Table S2. Additional references are cited only for contextual purposes and are not counted as core evidence sources. Relevant items are also noted in Supplementary Table S1 where applicable. No review protocol was registered, consistent with a critical narrative rather than systematic design.

### 2.6. Single-Author Process and Bias Safeguards

As noted above, this single-author critical narrative review did not use duplicate independent screening or extraction. The main safeguards against selective emphasis were therefore procedural transparency, predefined source-prioritization, consistent population-scope labeling of every source, source charting, a preference for primary evidence over reviews for numerical claims, transparent reporting of retained sources and retention counts (Supplementary Tables S1 and S2), and conservative, association-not-causation language for observational and claims-based findings. These safeguards are intended to make the basis of source selection and interpretation visible to the reader rather than to substitute for duplicate independent screening.

### 2.7. Ethics and Data Availability

Ethics approval and informed consent were not required because this review uses published literature and does not involve new patient-level data collection or analysis. All evidence discussed is derived from published sources cited in the reference list.

## 3. Results

Table 1, Table 2 and Table 3 summarize the pathway-stage cost drivers, intervention and pathway-value levers, and methodological safeguards used to interpret the evidence.

**Figure 2:**
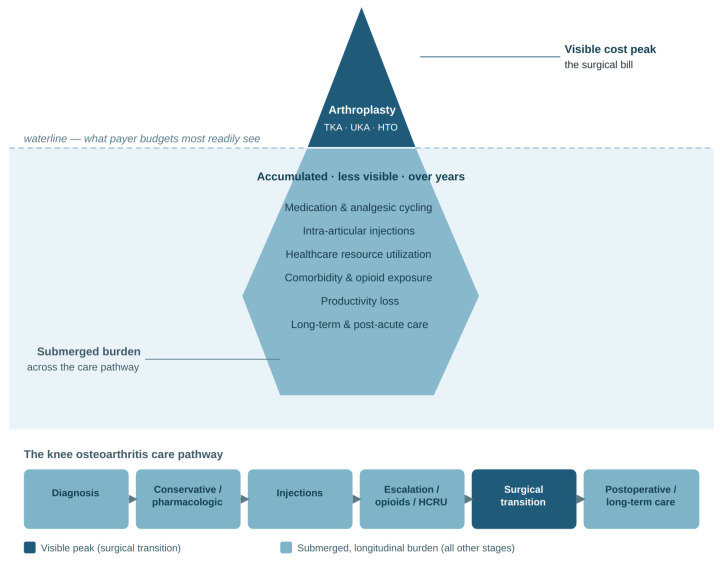
The economic iceberg of the knee osteoarthritis (KOA) care pathway.

Arthroplasty (total knee arthroplasty [TKA], unicompartmental knee arthroplasty [UKA], and high tibial osteotomy [HTO]) is the visible cost peak that payer budgets most readily see, whereas the less-visible burden accumulates across the pathway as years of medication and analgesic cycling, intra-articular injections, healthcare resource utilization, comorbidity and opioid exposure, productivity loss, and long-term care. The lower strip shows the six pathway stages; the surgical-transition stage corresponds to the visible peak and all other stages to the submerged, longitudinal burden. Figure 2 is conceptual and illustrative; supporting quantitative evidence is presented in the Results and tables.

### 3.1. Epidemiologic Scale, Time-in-Disease, and Cost Concentration

Knee osteoarthritis combines population-level scale with individual-level chronicity, and the resulting economic footprint is unusually concentrated. Global Burden of Disease 2019 estimates place prevalent KOA at approximately 364.6 million cases worldwide [9], and population-based syntheses report a comparable order of magnitude among adults aged 40 years and older [10] (Supplementary Table S1). This prevalence reflects a sustained disease state rather than a transient one: decision-analytic modeling of symptomatic KOA indicates a mean of roughly 28.4 years lived with the disease [11], so per-patient costs accrue across decades rather than within a single treatment episode. Lifetime symptomatic-disease risk is high and rises further with obesity and prior knee injury [13], leaving an at-risk denominator that is both large and expanding (per-source figures in Supplementary Table S1).

Against this background of scale and duration, measured spending is heavily concentrated in surgery. In the largest claims analysis identified, knee arthroplasty was performed in approximately 8.8% of patients yet accounted for 61.5% of KOA-related costs over the observation period, whereas hyaluronic acid represented 3.0% of overall costs [14]. In that claims analysis, the remaining measured KOA-related spending was distributed across nonsurgical outpatient, medication, injection, and other utilization categories; separate cost-of-illness and workforce studies show that productivity loss adds an additional societal layer to the pathway burden [15,16] (Supplementary Table S1). Newly diagnosed patients also incurred measurably higher all-cause costs than matched controls, beginning well before any surgical decision [12] (Table 1).

**Table 1:** KOA care-pathway cost drivers and evidence families.

Pathway Stage	Principal Cost Drivers	Dominant Evidence Scope/Population Label	Representative Evidence	Economic Signal (Direction/Scale)	Key Caveat
1—Diagnosis/early KOA	Primary-care visits, imaging, first-line analgesic/NSAID initiation	KOA·claims	Bedenbaugh 2021 [12]; Ong 2020 [14] (US)	Low per patient, high-volume; newly diagnosed +$7707 PPPY vs. matched controls [12]; cost accrues years before surgery	Stage misclassification—coding cannot grade radiographic/symptomatic severity
2—Conservative & pharmacologic care	Chronic NSAID/analgesic use, GI/CV adverse-event costs, physical therapy, weight management	KOA·claims + K/H-OA (supportive)	Taqi 2025 [17] (UK); Silverman 2022 [18] (US; K/H-OA, supportive)	Persistent NSAID use 27.0% at 1 yr [17]; oral NSAIDs incurred higher 36-month costs than topical NSAIDs [18].	Prescribing ≠ consumption; all-cause vs. KOA-attributable attribution
3—Intra-articular injections	IA-CS, IA-HA (product cost), PRP (often out-of-pocket), procedure visits	KOA·claims/KOA·model	Altman 2015 [19]; Mackowiak 2020 [20] (US)	Delay-to-arthroplasty association (median 484 vs. 114 d [19]); comparative injection-pathway costs were price-sensitive [20].	Confounding by indication; immortal-time bias; commercial evidence density (Supplementary Table S1)
4—Escalation opioids HCRU	Opioid prescribing, falls/fractures, ED and inpatient utilization, productivity loss	KOA·model/claims + OA-wide (supportive)	Huizinga 2022 [21]; Taqi 2021 [22]; Zhao 2019 [23] (US; OA-wide, supportive)	Lifetime opioid-related cost $14.0B (53% direct) [21]; falls aHR 2.40 [22].	OA-wide vs. KOA scope; associations, not causal effects
5—Surgical transition	TKA/UKA/HTO procedure and implant costs, pre-operative spending	KOA·claims/KOA·model	Bedard 2017 [24]; Konopka 2015 [25]; Smith 2017 [26]; Ruangsomboon 2025 [27]; Maman 2024 [28]	Concentrated cost peak—knee arthroplasty ~8.8% of patients = 61.5% of KOA-related cost [14]; HTO showed ~57% probability of cost-effectiveness at $50k/QALY [25].	Model-assumption dependence; perspective/conversion sensitivity; index ≠ lifetime
6—Postoperative long-term care	Post-acute rehabilitation, readmission, revision/conversion, persistent opioid use	KOA·claims	Courtney 2018 [29]; Navathe 2017 [30]; Leal 2022 [31] (UK)	90-day TKA episode median $23,800 [29]; bundling cut uncomplicated episodes −20.8% [30].	Incomplete downstream-event capture; bundled-payment risk adjustment

Note: Population labels per Supplementary Table S1 (KOA, knee osteoarthritis–specific; K/H-OA, knee and/or hip OA; OA-wide, osteoarthritis overall); non-KOA-specific evidence is supportive. Costs are reported in each source’s native currency and price year and are not pooled. PPPY, per-patient-per-year. Detailed numerical estimates and source-level limitations are provided in Supplementary Table S1.

Three cautions frame this concentration signal (Table 3). It is descriptive rather than causal: a high surgical-cost share indicates where spending is recorded, not that earlier-stage care is economically minor. Several underlying estimates also combine all-cause and KOA-attributable costs, which can over- or under-state the disease-specific burden depending on attribution. Finally, the headline concentration estimate derives from an industry-funded claims analysis [14]; its funding context is therefore reported transparently in Supplementary Table S1 and interpreted using the commercial-evidence safeguards summarized in Table 3, alongside the wider cost-distribution literature rather than as a stand-alone pathway summary.

### 3.2. Guideline Landscape and Real-World Discordance

Major clinical guidelines converge on the foundations of nonsurgical KOA care but diverge precisely at the interventions that drive discretionary spending. Recommendations from the American College of Rheumatology/Arthritis Foundation, OARSI, AAOS, and ESCEO consistently endorse exercise, weight management, and topical or oral NSAIDs as core therapy, and most advise against routine opioid use [3,4,5,6,8]. They differ, however, on intra-articular hyaluronic acid (IA-HA) and platelet-rich plasma (PRP), whose status ranges from conditional recommendation to recommendation against across societies, and several guidelines explicitly note the need for stronger cost-effectiveness evidence on injectable therapies [4,5,6] (Table 2).

The economically consequential gap lies between recommendation and practice. A systematic review of guideline quality reported that fewer than 40% of patients receive guideline-concordant care [7], and national practice surveys describe heterogeneous, non-standardized management in which injectable therapies—including IA-HA—are frequently preferred despite their conditional guideline status and limited analgesic satisfaction [32,33]. This discordance is economically important because it generates predictable low-value spending nodes, most notably repeated IA-HA use late in the pathway and close to eventual surgery. Two cautions follow (Table 3): recommendation strength does not equate to real-world feasibility or uptake, and the commercially dense IA-HA evidence base is relevant to interpreting the guideline–practice divergence discussed in Section 3.5.

### 3.3. Analgesic Utilization, NSAID Constraints, and Oral-Therapy Cost-Effectiveness

Analgesic management of KOA is multi-class and persistent rather than sequential and time-limited. Large primary-care cohort data show that, after analgesic initiation, a substantial proportion of patients remain on antidepressants, NSAIDs, or opioids one year later, and that opioid and tramadol use in particular has increased over time [17,34]. High utilization is nonetheless constrained by comorbidity and contraindication: in population-register data on knee/hip OA (supportive evidence, not KOA-specific), roughly one in five patients used NSAIDs despite a documented contraindication, and contraindicated patients were correspondingly more likely to receive opioids [35]. Trial-level synthesis in knee/hip OA likewise favors topical and oral NSAIDs over opioids for pain while assigning opioids a markedly worse adverse-event profile [36] (Supplementary Table S1).

Critically, high or prolonged use cannot be read as evidence of effectiveness. Long-term NSAID exposure shows only modest or non-significant symptom benefit in observational cohorts, and apparent deterioration among sustained users more plausibly reflects confounding by severity than treatment failure [37,38] (Table 3). The cost-effectiveness of oral therapy is correspondingly conditional rather than fixed, being highly sensitive to comparator, drug price, and gastrointestinal or cardiovascular toxicity. Inexpensive agents can be cost-saving in appropriate patients, whereas higher-priced or safety-constrained regimens yield incremental cost-effectiveness ratios well above conventional thresholds—on the order of $284,630 per QALY for generic celecoxib in high-pain modeling [39]—with wide ICER ranges across drug classes reported in OA-wide cost-effectiveness reviews, used here as supportive rather than KOA-specific economic evidence [40] (Table 2). No oral analgesic therefore holds a context-free economic ranking; the value depends on the patient, the price, and the comparator.

### 3.4. Opioid Exposure as a Pharmacoepidemiologic and Economic Risk Signal

Across utilization studies, opioid exposure in KOA behaves less like an inexpensive analgesic and more like a marker of severity, comorbidity, and downstream cost. Claims series consistently show appreciable and historically rising opioid use, often increasing as patients approach surgery [41,42,43,44], with broadly concordant patterns in supportive knee/hip OA and general-OA data (not KOA-specific) [23,45,46]. The economic relevance of this exposure is threefold. First, it carries safety-linked costs: current opioid use was associated with a markedly higher short-term fall risk (adjusted hazard ratio of 2.40 in the first six months after diagnosis) [22], and falls are cost-generating events themselves. Second, in KOA-specific modeling, the cumulative burden is large and substantially indirect—an Osteoarthritis Policy Model analysis estimated a lifetime opioid-related cost of $14.0 billion in the U.S. symptomatic-KOA population, of which only 53% was direct medical costs, with the remainder reflecting lost productivity and other societal costs [21]. Third, opioid exposure bridges nonsurgical management to the surgical episode: preoperative opioid use increased 90-day arthroplasty episode costs by $789 [47] (its downstream post-acute cost implications are quantified in Section 3.8).

The consistent caution across these data is that the relationship is associative, not causal (Table 3): opioid use marks more severe and more comorbid disease, so the higher accompanying costs partly reflect who is treated rather than the drug itself. Interpreted accordingly, the evidence supports treating opioid exposure as a high-risk, high-cost utilization signal rather than a low-cost option, without asserting a causal effect of opioid spending per se. Drug-level prevalence, temporal trends, and per-patient cost differences are reported in Supplementary Table S1.

### 3.5. Injection Pathway: IA-HA and PRP as Economic Decision Nodes

Intra-articular therapy is where guideline ambiguity, real-world demand, and commercial activity converge, making injections distinct economic decision nodes rather than incidental costs. For intra-articular corticosteroid, repeated use carries a structural caution: a controlled trial found greater cartilage loss without a significant pain advantage over two years [48], so its low unit cost must be weighed against limited durable benefit. The larger economic question concerns intra-articular hyaluronic acid (IA-HA), which is repeatedly associated in administrative data with a longer observed time to arthroplasty—for example, a median time to knee replacement of 484 versus 114 days in treated versus untreated patients, with a course-count gradient reported in claims data [19], and concordant delay signals in other claims cohorts [49,50,51]. This association is central to IA-HA’s economic case and is also its principal interpretive hazard: because patients must remain replacement-free to continue receiving injections, the apparent delay is strongly susceptible to immortal-time bias, confounding by indication, and disease-stage misclassification (Table 3), and IA-HA value is in any case stage-dependent rather than uniform.

A further consideration applies specifically to this evidence base. The IA-HA economic literature was commercially dense, with several product-level or claims-based analyses funded by or involving manufacturers; therefore, the repeated delay-to-TKA signal was interpreted as a real-world association requiring caution rather than as definitive causal evidence. The funding and conflict-of-interest status of these sources is documented individually in Supplementary Table S1. Platelet-rich plasma (PRP) presents a different economic profile: its cost-effectiveness is highly sensitive to price and durability—favorable only below a low per-course cost threshold in decision modeling (on the order of $1192 over twelve months) [52]—and it remains guideline-discordant and protocol-heterogeneous. Notably, the PRP evidence is not uniformly industry-funded, although several analyses carry orthobiologics-related author ties (Supplementary Table S1). Delay magnitudes, injection cost-effectiveness ratios, product-level comparisons, and per-source funding status are reported in Supplementary Tables S1 and S2.

Broader review-level evidence on IA-HA similarly emphasizes heterogeneity in products, comparators, and methodological assumptions, supporting the stage- and bias-aware interpretation adopted here [53].

### 3.6. Real-World HCRU, Multimorbidity, and Cross-System Burden

KOA is associated with substantial healthcare resource utilization (HCRU), but the measured cost signal reflects both knee-specific care and the comorbidity, pain severity, and access patterns that accompany the diagnosis. In KOA-specific U.S. Medicare data, mean total cost reached $15,558 per patient—$5364 higher than matched controls [54]—and across health systems the disease consistently raises utilization above comparator populations. In supportive knee/hip OA data (not KOA-specific), linked primary-care and hospital records showed annual costs roughly five-fold higher in affected patients than controls (£4199 versus £781), accompanied by extensive therapy-line cycling [55]. Two patterns recur across these heterogeneous sources. First, comorbidity and contraindication concentrate cost—utilization is highest in subgroups such as patients with NSAID contraindications—so a substantial fraction of measured spending is comorbidity-related rather than strictly KOA-attributable [56]. Second, indirect costs are not a minor addendum: in several societal-perspective studies, productivity loss and informal care exceed direct medical costs, underscoring that payer-only accounting understates the pathway burden [57,58] (Supplementary Table S1).

These data are best read with two cautions (Table 3). Cross-country cost figures are reported in their native currencies and are not pooled, because reimbursement structure, currency, price year, and care organization differ; and elevated utilization may partly reflect access and comorbidity rather than KOA severity alone. Country-specific cost estimates, currency and price-year details, and the direct-versus-indirect split for each source are provided in Supplementary Tables S1 and S2.

### 3.7. Pre-Arthroplasty Spending and the Downstream Surgical Cost State

Two features define the surgical end of the pathway: spending rises sharply before arthroplasty, often on guideline-discordant care, and the downstream procedure choice has no fixed economic ranking. In the year before total knee arthroplasty, nonarthroplasty treatments accounted for a majority of non-inpatient KOA cost (57.6% in a large claims cohort), of which intra-articular hyaluronic acid alone represented 29.3% despite its limited guideline support, while strongly recommended interventions made up a much smaller share [24]. This pre-surgical window therefore contains substantial potentially low-value spending close to the point of surgery, reinforcing the injection-economics caution of Section 3.5.

The mid-stage surgical decision between joint-preserving and arthroplasty options illustrates why downstream value is scenario-dependent. For medial-compartment disease, comparative economic estimates for high tibial osteotomy (HTO) and unicompartmental knee arthroplasty (UKA) derive from decision-analytic models that differ in perspective, time horizon, and assumptions about reoperation and conversion to TKA. In a U.S. lifetime societal model, HTO and UKA produced nearly identical discounted QALYs while direct medical costs favored HTO, giving HTO a 57% probability of being cost-effective at a $50,000/QALY threshold [25]; an age-stratified 10-year model instead favored HTO below and UKA above approximately 60 years of age [26], and a Canadian public-payer model favored UKA outright [27]. Because jurisdiction, model structure, time horizon, and downstream-event definitions can reverse the ranking, HTO and UKA are best treated as context-sensitive options for selected patients rather than as a fixed hierarchy. Joint-preserving alternatives, including proximal fibular osteotomy, remain part of the selected-patient procedure-mix discussion but are not central to the economic ranking examined here; procedure-trend and comparative-surgery evidence are interpreted alongside contextual reviews rather than used as primary economic support [59,60]. A broader comparative narrative synthesis of HTO–UKA economics is retained only as contextual background [61]. Primary cohort data on medial open-wedge HTO additionally inform its complication, reoperation, and survivorship profile [62].

Index-procedure comparisons require the same caution (Table 3). UKA is associated with shorter length of stay and lower index hospitalization cost than TKA, and facility costs fall further in outpatient and ambulatory-surgery settings; these are index-admission figures that omit revision risk, conversion pathways, implant survival, and patient selection, and are reported in Supplementary Tables S1 and S2 rather than read as a complete long-term ranking. TKA remains the dominant endpoint for advanced disease, so the pathway-relevant question is how earlier medication, injection, and rehabilitation choices affect the probability, timing, type, and cost of eventual arthroplasty—not whether arthroplasty is simply expensive.

### 3.8. Post-Arthroplasty Utilization, Perioperative Drug Use, and Bundled-Payment Policy

The arthroplasty episode is costly but substantially modifiable, which is why it has become the focus of bundled-payment policy. Medicare data place the median 90-day total knee arthroplasty episode at roughly $23,800, with wide variation driven by patient risk [29], and observational bundled-payment evidence shows that uncomplicated episode costs can fall by about one-fifth (from $26,785 to $21,208), achieved largely through lower implant prices and reduced post-acute spending rather than through fewer services alone [30]. Post-acute care is therefore a principal cost lever, and preoperative status is linked to post-acute spending in observational data: as introduced in Section 3.4, preoperative opioid exposure was associated with roughly 70% higher post-acute care costs [47], connecting upstream medication patterns to downstream episode spending. The perioperative period itself remains pharmacologic, with NSAIDs and opioids dominating in-hospital pain-management drug expense [63] (Supplementary Table S1).

**Table 2:** Intervention and pathway-value levers in KOA.

Intervention/Value Lever	Stage/Indication	Population Scope	Guideline Positioning/Discordance	Economic-Value Signal	Value Conditions	Main Interpretive Caution
Exercise rehabilitation weight management	All stages; foundational	K/H-OA (supportive)	Strongly recommended (ACR/AF, OARSI)	Foundational low-cost, guideline-supported care; real-world uptake incomplete (~57% attending PT in supportive K/H-OA data [35]).	Value realized with adherence and sustained programs	Real-world under-use; adherence
Topical NSAIDs	Early–moderate	K/H-OA (supportive)	Recommended (OARSI: topical strong)	Favorable safety-cost profile: +$689 topical vs. +$4812 oral over 36 mo [18].	Most valuable in older/comorbid patients	Under-used relative to oral; efficacy ceiling
Oral NSAIDs	Moderate	KOA·model + K/H-OA (supportive)	Conditionally recommended with GI/CV caution	Effective but toxicity- and comparator-sensitive; generic celecoxib high-pain ICER $284,630/QALY [39].	Cost-saving for inexpensive agents in low-risk patients; poor value at high price or high CV/GI risk	Used despite contraindications—~21% with a contraindication still used NSAIDs [35]
Opioids	Escalation	KOA·model/KOA·claims	Generally discouraged; tramadol only conditionally/rescue in selected settings.	Net-negative value profile; preoperative opioid use linked to higher 90-day arthroplasty episode cost [47].	No identified context of net economic value; risk rises with persistence	Association not causal; severity confounding
IA corticosteroids (IA-CS)	Moderate flare	KOA·sx/KOA·claims	Conditionally recommended	Short-term, low unit cost (~$1230 4-yr monotherapy PPPM [20]).	Value for short-term flare control; not for repeated long-term dosing	Transient effect; repeated triamcinolone → greater cartilage loss, no significant pain advantage [48]
IA hyaluronic acid (IA-HA)	Moderate	KOA·claims	Discordant recommendations: restrictive in ACR/AAOS, more permissive in selected OARSI/ESCEO contexts; widely used in real-world claims.	Delay-to-TKA association (TKA-free survival 85.8% vs. 74.1% at 1 yr [51]); price-sensitive.	More favorable used earlier and with higher-molecular-weight products; value uncertain late or near surgery	Confounding; immortal-time bias; funding/COI context reported in Supplementary Table S1.
Platelet-rich plasma (PRP)	Moderate	KOA·model	Discordant recommendations: insufficient guideline support vs. variable self-pay use.	Price/durability-sensitive; cost-effective only below ~$1192/12 mo [52]; immediate TKA dominated PRP in a payer model [64].	Value only when all-in price is low, utility gain meaningful, and durability sufficient	Heterogeneity; durability; funding/COI context reported in Supplementary Table S1.
High tibial osteotomy (HTO)	Younger, medial unicompartmental disease	KOA·model + KOA·clin (primary [62])	Option in selected phenotypes	~57% probability of cost-effectiveness at $50k/QALY; favored below ~60 y [25,26].	Value in younger, active, medial-compartment patients	Model-assumption dependence; conversion-to-TKA risk; complication/survivorship [62]
Unicompartmental knee arthroplasty (UKA)	Older/selected unicompartmental disease	KOA·model/KOA·claims	Option in selected phenotypes	Most cost-effective in some models (55.3% of simulations [27]); lower index hospitalization cost than TKA [28].	Value with appropriate selection; outpatient/ASC settings lower facility cost	Revision risk; patient selection; index ≠ lifetime
Total knee arthroplasty (TKA)	End-stage	KOA·model/KOA·claims	Recommended for end-stage disease	High upfront cost; net societal benefit $18,930/patient in a societal model [65]; dominant downstream cost state.	Value established for advanced disease; depends on perioperative/post-acute management	Cost peak; perioperative and post-acute burden
Pre-TKA nonoperative treatment cycling (value lever)	Late, pre-surgical	KOA·claims	Frequently guideline-discordant; much late pre-TKA spend on conditionally/non-recommended care.	Pre-TKA nonarthroplasty care 57.6% of non-inpatient cost; guideline-concordant use could reduce cost by ~45% [24].	Value depends on appropriate symptom management vs. futile treatment cycling near surgery	Observational; timing relative to surgery; severity confounding
Bundled payment/post-acute coordination (value lever)	Post-surgical/system-level	KOA·claims	Policy mechanism (e.g., CMS bundles), not a clinical guideline	Uncomplicated episode costs fell by about one-fifth under bundling, mainly via lower implant and post-acute spending [30].	Savings realized only with adequate risk adjustment and post-acute inclusion	Unadjusted models can reward selection away from complex patients

Note: Guideline positioning summarizes major society recommendations (ACR/AF; OARSI; AAOS, which flags the need for stronger injectable-therapy cost-effectiveness evidence; ESCEO stepwise algorithm); “discordance” denotes a gap between recommendation and observed real-world utilization. Economic-value signals are reported in native currency; perspectives, currency/cost year, and funding/COI status appear in Supplementary Table S1. Population labels as in Table 1; non-KOA-specific rows are supportive. The final two rows are pathway-value levers rather than single interventions. Additional source-level numerical estimates and limitations are provided in Supplementary Table S1.

**Table 3:** Methodological challenges in KOA pharmacoeconomic evidence and the interpretive safeguards applied.

#	Methodological Challenge/Bias	Where It Arises (Evidence Family Typical Design)	Effect on Economic Interpretation	Interpretive Safeguard Applied in This Review
1	Confounding by indication/channeling	Drug- and injection-utilization studies · observational claims/registry	Treated patients differ systematically from untreated; apparent cost/outcome differences may reflect who was selected, not the intervention	Findings reported as real-world associations, not causal effects; indication and channeling noted; within-design comparisons preferred
2	Immortal-time/time-dependent exposure bias	Delay-to-arthroplasty analyses (e.g., IA-HA “delays TKA”) · longitudinal claims	Patients must remain event-free to accrue exposure, artificially inflating apparent time-to-surgery and the value of the intervening treatment	Flagged explicitly; the IA-HA delay signal is interpreted as an association limited by immortal-time bias rather than a causal delay
3	Cost-attribution ambiguity (all-cause vs. disease-specific)	HCRU and cost-burden studies · claims	All-cause costs overstate KOA-attributable burden; attributable-only costs may understate it	Attribution basis distinguished (all-cause vs. OA-related vs. KOA-attributable vs. treatment-specific) and stated alongside each estimate
4	Population-scope mismatch (OA-wide vs. knee/hip vs. KOA)	Burden, utilization, and NSAID/opioid studies	General-OA or knee/hip estimates are read as KOA-specific, blurring the target population	Every source carries a population label (KOA/K/H-OA/OA-wide; Supplementary Table S1); non-KOA-specific evidence flagged “supportive” at first mention
5	Disease-stage misclassification	Claims/registry studies lacking radiographic or symptomatic grading	Early and late KOA economics are pooled, obscuring stage-dependent value	Stage limitations stated; medication, injection, and surgical value interpreted as stage- and phenotype-dependent
6	Analytic-perspective heterogeneity	Cost-effectiveness and cost studies	Narrow payer perspectives omit productivity and patient costs; societal and payer results are not comparable	Perspective recorded for each source (Supplementary Table S1); estimates from different perspectives are not equated
7	Currency, cost-year, and costing-basis variation	Cross-country cost and CEA studies	Naïve cross-country cost comparison is misleading once currency, price year, and costing differ	Costs retained in native currency and price year without conversion or inflation adjustment; currency, year, country, and costing basis reported
8	Limited cross-system transferability	US-claims-dominated evidence base	Reimbursement structure, pricing, and care organization limit export of findings to other systems	Cross-country results interpreted qualitatively rather than pooled; transferability and the US-centricity of the evidence base discussed explicitly
9	Commercial/funding influence and evidence density	IA-HA and PRP literature · industry-funded evaluations	High evidence volume in commercially active areas can be mistaken for evidence strength or pathway priority	Funding source and conflicts of interest flagged (Supplementary Table S1); funding used as interpretive caution, not exclusion; evidence volume not treated as evidence strength
10	Model-structure and assumption dependence	Decision-analytic models (e.g., HTO/UKA/TKA CEA)	Results hinge on conversion rates, time horizon, discounting, and input sourcing; a single “winner” can be an artifact of assumptions	Model horizon and key assumptions recorded (Supplementary Table S1); HTO–UKA economics framed as scenario- and age-dependent rather than a context-free hierarchy
11	Selective-inclusion risk in a single-author narrative	The review process itself	A broad single-author review risks emphasizing convenient or confirmatory evidence	Predefined source-prioritization, primary-over-review preference for numerical claims, and transparent reporting of retained sources/counts (Supplementary Tables S1 and S2)
12	Evidence-volume (vote-counting) fallacy	Synthesis across heterogeneous studies	Counting concordant studies misrepresents weight when designs and populations differ	Conflicting findings weighed by design strength, population match, perspective, time horizon, and completeness of downstream-event capture—not tallied
13	Incomplete downstream-event capture/informative censoring	Longitudinal claims, registry, perioperative, bundled-payment, and model studies	Short follow-up or incomplete capture of falls, adverse events, complications, revision, conversion to TKA, post-acute care, or persistent opioid use can underestimate cumulative pathway costs or overstate short-term value	Time horizon, censoring, and downstream-event capture are stated when available; short-window findings are interpreted as partial pathway evidence rather than complete value estimates

Note: Safeguards are interpretive, not a formal risk-of-bias assessment; consistent with a critical narrative rather than systematic-review design (Section 2). “IA-HA,” intra-articular hyaluronic acid; “PRP,” platelet-rich plasma; “HCRU,” healthcare resource utilization; “CEA,” cost-effectiveness analysis; “KOA,” knee osteoarthritis; “K/H-OA,” knee and/or hip osteoarthritis; “TKA/UKA/HTO”, total knee arthroplasty/unicompartmental knee arthroplasty/high tibial osteotomy.

Two cautions bound these figures (Table 3). Bundled-payment savings depend heavily on risk adjustment, because unadjusted models can reward selection away from complex patients rather than genuine efficiency; and episode costs are meaningful only when post-acute care, readmission, and revision are included rather than truncated at discharge. Episode-cost distributions, savings components, and post-TKA outpatient utilization are reported in Supplementary Tables S1 and S2.

### 3.9. Methodological Challenges and Conditional Value Synthesis

The preceding sections share a single methodological lesson: KOA economic estimates are conditional, and apparent contradictions across studies often reflect how each study was designed rather than genuine disagreement (Table 3). Four cautions recur and should govern interpretation. First, short follow-up underestimates pathway cost, because falls, persistent opioid use, complications, revision, and conversion to surgery accrue over years rather than months. Second, associations from claims and registry data are not causal—the IA-HA delay-to-arthroplasty signal is the clearest example, limited by immortal-time bias and confounding by indication. Third, commercial evidence density can distort apparent pathway priority: a heavily studied, manufacturer-involved intervention area is not thereby a high-value one, and funding or conflict-of-interest status was used here as an interpretive caution rather than as an exclusion criterion. Fourth, differences in population scope, analytic perspective, time horizon, and costing basis explain most cross-study divergence, so estimates from different designs should not be equated or pooled.

Viewed through these cautions, the evidence points to a small set of decision-relevant levers rather than a single cost-saving intervention: prioritizing early guideline-concordant conservative care, minimizing opioid exposure, using injections in a stage-appropriate way, optimizing patients before surgery, and structuring arthroplasty bundles with adequate risk adjustment. None of these is established by any single study; each follows from the pathway-level pattern that value in KOA depends on stage, perspective, horizon, and comparator. The specific biases noted here, with the safeguards applied in this review, are cataloged in Table 3 and are not restated individually in this section.

## 4. Discussion

### 4.1. Knee Osteoarthritis as a Long-Horizon Utilization Pathway

The central implication of this review is that the economics of KOA cannot be captured by any single cost—least of all the price of surgery. The evidence assembled here describes a disease that unfolds over decades, accumulating cost through diagnosis, conservative and pharmacologic care, injections, escalation and opioid exposure, surgical transition, and post-acute care (Table 1 and Table 2). Within that trajectory, measured spending is concentrated in the surgical state, yet that surgical bill is the visible tip of a less-visible, partly indirect, longitudinal burden. Reframing KOA economics in pathway terms changes the unit of analysis from the procedure to the patient trajectory, and it reframes the policy question from “how much does arthroplasty cost?” to “how do earlier choices shape the probability, timing, type, and cost of everything downstream?”.

### 4.2. Guideline Discordance and Implementation Gaps as Economic Mechanisms

A recurring theme is that the gap between guideline-recommended care and real-world practice is not merely a quality problem but an economic mechanism. Guidelines converge on low-cost foundational care and diverge mainly on injectable therapies (Table 2), yet meaningful real-world spending can flow to conditionally recommended or non-recommended interventions, particularly late in the pathway and close to surgery. Discordance therefore creates identifiable low-value spending nodes that are, in principle, addressable without restricting effective care. Crucially, recommendation strength is not the same as feasibility or uptake; narrowing the implementation gap requires attention to access, adherence, and incentives, not only to what guidelines state.

### 4.3. Medication Exposure, Especially Opioids, as a Risk-and-Cost Signal

The pharmacoepidemiologic evidence reframes analgesic use from a low-cost early step to a marker of severity, comorbidity, and downstream cost. This is clearest for opioids, where exposure is best interpreted as a high-risk utilization signal—associated with falls, productivity loss, and higher surgical-episode costs—rather than as an inexpensive analgesic alternative (Section 3.4, Table 2). The consistent interpretive caution is that these are associations: opioid use marks patients who are sicker and more complex, so the accompanying costs partly reflect who is treated rather than the drug itself. The economic argument for minimizing avoidable opioid exposure therefore rests less on pharmacy spending than on the downstream consequences that exposure signals.

### 4.4. Injections: Stage-Dependent and Bias-Aware Interpretation

Intra-articular therapy is the clearest illustration of why KOA economic evidence must be read with methodological caution. The repeated association between IA-HA and a longer observed time to arthroplasty is present in the data but is strongly susceptible to immortal-time bias and confounding by indication, and so cannot support a causal “delay” claim (Section 3.5, Table 3). Two further features complicate interpretation: the value of injections appears stage-dependent rather than uniform, and the evidence base is commercially dense, with several product-level or claims-based analyses funded by or involving manufacturers (documented in Supplementary Table S1). Treating commercial density as a reason for interpretive caution—rather than as either automatic disqualification or endorsement—is essential to avoid mistaking evidence volume for evidence strength.

### 4.5. Arthroplasty as the Visible Cost State, Not the Whole Burden

Arthroplasty is the dominant downstream cost state, but its economics are neither fixed nor self-contained. The value of surgery depends on the preoperative pathway, procedure selection, post-acute care, and risk-adjusted payment design, and procedure-level comparisons—for example, high tibial osteotomy versus unicompartmental arthroplasty—are scenario-dependent rather than universal, varying with age, perspective, time horizon, and jurisdiction (Section 3.7). Bundled-payment evidence shows that episode costs are modifiable, but realized savings depend on adequate risk adjustment and on counting post-acute care within the episode (Section 3.8). The pathway-relevant lesson is that surgery should be evaluated as a downstream state shaped by upstream decisions, not as a stand-alone expense.

### 4.6. Transferability Across Health Systems

Because the evidence base is dominated by U.S. claims data and is heterogeneous in currency, cost basis, perspective, and care organization, its findings transfer across health systems only with caution. This is why cost estimates were retained in their native currency and price year and were not pooled (Section 2.3, Table 3): a U.S. payer-episode cost, a UK linked-records estimate, and a tariff-based analysis from another system are not interchangeable, and naïve comparison would manufacture false precision. Readers in other systems should treat the directional patterns—concentration of spend in surgery, pre-surgical low-value cycling, opioid risk, and stage-dependent injection value—as more transferable than the absolute figures, and should anchor local decisions in local cost and reimbursement data.

### 4.7. Implications for Clinical Practice, Policy, and Future Research

For clinical practice and policy, the pathway view supports a small set of decision-relevant levers rather than any single cost-saving intervention—prioritizing early guideline-concordant conservative care, minimizing avoidable opioid exposure, using injections in a stage-appropriate and cost-aware way, optimizing patients before surgery, and designing arthroplasty bundles with adequate risk adjustment (Section 3.9). The most decision-relevant research gaps follow directly from the methodological limitations identified throughout. Future work should link claims, registry, and patient-reported data so that the full pathway—rather than isolated episodes—is captured; report directly measured, preference-based utilities and propagate utility and mapping uncertainty through sensitivity analysis, given how often KOA economic conclusions hinge on small QALY differences; design delay-to-arthroplasty analyses that explicitly address immortal-time bias and confounding by indication; separate KOA-specific from broader-OA populations and early- from late-stage disease; and routinely report productivity and societal costs alongside payer perspectives. Independent, non-industry economic evaluations of commercially dense interventions and head-to-head analyses of injection strategies and joint-preserving versus arthroplasty options in well-defined phenotypes would be especially valuable.

### 4.8. Strengths and Limitations

The principal strength of this review is its pathway-level, critically appraised synthesis of heterogeneous pharmacoeconomic and pharmacoepidemiologic evidence, organized so that population scope, study design, analytic perspective, and funding context are made explicit (Section 2.4, Table 1, Table 2 and Table 3 and Table S1). The work is also subject to limitations inherent to its design. As a critical narrative—rather than systematic—review, it did not use a registered protocol, duplicate independent screening, formal risk-of-bias scoring, or quantitative pooling, and a single-author process carries a risk of selective emphasis; these were mitigated through predefined source prioritization, consistent population-scope labeling, a preference for primary over review sources for quantitative claims, transparent reporting of retained sources, and conservative association-not-causation language (Section 2.4, Section 2.5 and Section 2.6). The evidence base itself is US-centric, heterogeneous in cost and perspective, and commercially dense in several intervention areas, which constrains both pooling and cross-system transferability; consistent with this, costs were deliberately retained in native currency rather than converted. Finally, because much of the underlying evidence is observational, the review characterizes economic associations and pathway-level patterns rather than establishing causal effects of specific treatments on cost.

## 5. Conclusions

Knee osteoarthritis should be evaluated as a longitudinal care pathway rather than as a surgically defined cost. Value depends on disease stage, treatment exposure, comorbidity, analytic perspective, and complete capture of downstream events, including post-acute care and revision. Value-based KOA policy should therefore link claims, registries, patient-reported outcomes, preference-based utilities, and productivity data to measure cumulative value beyond the arthroplasty episode alone.

## Data Availability

No new data were created or analyzed in this study.

## Supplementary Materials

The following supporting information can be downloaded at https://www.mdpi.com/article/10.3390/healthcare14142066/s1, Table S1: Retained core evidence sources used in the synthesis [66,67,68,69,70,71,72,73,74,75,76,77,78,79,80,81,82,83,84,85,86,87]; Table S2: Descriptive source-identification summary for the critical narrative review. Counts summarize how candidate sources were narrowed to the core evidence base used in the synthesis; File S1: Search Concepts, Representative Search Strings, Source-Prioritization Criteria, and Source-Charting Domains.

## Abbreviations

AAOS	American Academy of Orthopaedic Surgeons
ACR	American College of Rheumatology
BMI	body mass index
CAGR	compound annual growth rate
CDC	Centers for Disease Control and Prevention
CHECK	Cohort Hip and Cohort Knee study
CI	confidence interval
COPD	chronic obstructive pulmonary disease
COX-2	cyclooxygenase-2
CPRD	Clinical Practice Research Datalink
DDD	defined daily dose
EHR	electronic health record
EQ-5D	EuroQol 5-Dimension
EQ-5D-5L	EuroQol 5-Dimension 5-Level
ESCEO	European Society for Clinical and Economic Aspects of Osteoporosis, Osteoarthritis and Musculoskeletal Diseases
GBD	Global Burden of Disease
GDP	gross domestic product
HA	hyaluronic acid
HCRU	healthcare resource utilization
HIRA	Health Insurance Review and Assessment Service
HR	hazard ratio
HRQoL	health-related quality of life
HTO	high tibial osteotomy
IA-HA	intra-articular hyaluronic acid
ICER	incremental cost-effectiveness ratio
JPY	Japanese yen
KL	Kellgren–Lawrence grading scale
KOA	knee osteoarthritis
MCBS	Medicare Current Beneficiary Survey
MOST	Multicenter Osteoarthritis Study
NIS	National Inpatient Sample
NSAID	nonsteroidal anti-inflammatory drug
OA	osteoarthritis
OAI	Osteoarthritis Initiative
OAPol	Osteoarthritis Policy Model
OARSI	Osteoarthritis Research Society International
OR	odds ratio
OTC	over-the-counter
PPI	proton pump inhibitor
PRGF	plasma rich in growth factors
PRP	platelet-rich plasma
PT	physical therapy
QALY	quality-adjusted life year
RCT	randomized controlled trial
SF-6D	Short Form 6-Dimension
SKOA	symptomatic knee osteoarthritis
SYSADOA	symptomatic slow-acting drug for osteoarthritis
THA	total hip arthroplasty
TKA	total knee arthroplasty
TKR	total knee replacement
UK	United Kingdom
UKA	unicompartmental knee arthroplasty
US	United States
USD	United States dollar
WOMAC	Western Ontario and McMaster Universities Osteoarthritis Index
YLD	years lived with disability
