# Supplementary material for: Beyond the Surgical Bill: Pharmacoeconomics and Real-World Utilization Across the Knee Osteoarthritis Care Pathway—A Critical Narrative Review
Source: Healthcare (Basel). 2026 Jul 9;14(14):2066. doi: 10.3390/healthcare14142066 (PMC13409983; doi:10.3390/healthcare14142066)
Supplement: Supplementary file 1 [file healthcare-14-02066-s001.zip › Supplementary File S1.pdf]

Supplementary material File S1

Search Concepts, Representative Search Strings, Source-Prioritization Criteria, and Source-Charting Domains

This document describes the structured source-identification procedure summarized in the Methods. It is provided to support transparency for a critical narrative review and is not intended to imply a systematic-review eligibility process; no duplicate independent screening, formal risk-of-bias scoring, or quantitative pooling was performed.

1. Databases and Search Dates

Four sources were used to identify relevant literature: PubMed, Web of Science, Scopus, and Google Scholar. PubMed served as the primary biomedical database for indexed clinical, pharmacoepidemiologic, and health-economic studies. Web of Science and Scopus supported citation tracking and cross-disciplinary identification of economic and health-services research. Google Scholar was used as a supplementary, relevance-ranked tool (see A.3).

Publications issued between 1 January 2000 and 30 April 2026 were considered. Searches were performed between 1 April 2026 and 30 April 2026 across all four sources, with supplementary citation tracking. No geographic restrictions were applied; however, because cost estimates are highly sensitive to reimbursement structure, currency, price year, payer system, and site of care, cross-country results were interpreted qualitatively rather than pooled numerically.

2. Search Concepts

Searches combined knee-osteoarthritis (KOA) terms with pharmacoeconomic, pharmacoepidemiologic, utilization, and pathway terms.

Condition terms: “knee osteoarthritis”, “knee OA”, “gonarthrosis”, “hip and knee osteoarthritis”, “osteoarthritis”.

Economic and utilization terms: “pharmacoeconomics”, “pharmacoepidemiology”, “cost”, “cost of illness”, “economic burden”, “healthcare resource utilization”, “HCRU”, “claims”, “registry”, “real-world evidence”, “drug utilization”, “cost-effectiveness”, “cost-utility”, “QALY”, “ICER”, “decision model”, “Markov”.

Intervention and pathway terms: “NSAID”, “topical NSAID”, “opioid”, “tramadol”, “acetaminophen”, “paracetamol”, “duloxetine”, “intra-articular corticosteroid”, “hyaluronic acid”, “viscosupplementation”, “platelet-rich plasma”, “PRP”, “physical therapy”, “bracing”, “radiofrequency ablation”, “total knee arthroplasty”, “total knee replacement”, “unicompartmental knee arthroplasty”, “high tibial osteotomy”, “revision”, “post-acute care”, “bundled payment”, “glucosamine”, “chondroitin”, “SYSADOA”, “symptomatic slow-acting drugs for osteoarthritis”, “willingness-to-pay”, “net monetary benefit”, “budget impact”, “health technology assessment”, “price threshold”.

3. Representative Search String and Database Adaptation

A representative PubMed concept structure was:

(“knee osteoarthritis” OR “knee OA” OR gonarthrosis) AND (pharmacoeconomics OR pharmacoepidemiology OR cost OR “cost of illness” OR “economic burden” OR utilization OR

claims OR registry OR "real-world evidence" OR "drug utilization" OR "cost-effectiveness" OR "cost utility" OR QALY OR ICER OR Markov OR "decision model")

The exact syntax was adapted across databases because indexing and search operators differ between PubMed, Web of Science, Scopus, and Google Scholar. For Web of Science and Scopus, the same concept blocks were entered as topic/title–abstract–keyword queries and supplemented by forward and backward citation tracking of key economic and health-services sources.

Google Scholar (first-100 rationale). Google Scholar was used as a supplementary, relevance-ranked search tool rather than a primary systematic-search instrument. The first 100 results from the main combined search strategy were screened to identify economic models, recent publications, institutional full texts, and sources not consistently captured in indexed databases. This bound was adopted because relevance ranking declines substantially beyond the early results in broad narrative searches, so screening additional pages yields diminishing returns while disproportionately surfacing off-topic records.

4. Source-Prioritization Criteria

Sources were prioritized when they met one or more of the following criteria:

KOA-specific pharmacoeconomic, pharmacoepidemiologic, HCRU, or cost-effectiveness outcomes;

claims, registry, electronic-health-record, or administrative-database evidence relevant to real-world utilization;

drug-utilization evidence involving NSAIDs, opioids, acetaminophen/paracetamol, antidepressants, antiepileptic drugs, or perioperative analgesics;

injection-focused evidence involving intra-articular corticosteroids, intra-articular hyaluronic acid, PRP, or product-level injection heterogeneity;

economic evaluations reporting costs, QALYs, ICERs, net monetary benefit, or price-threshold findings;

evidence on pre-arthroplasty spending, arthroplasty episode costs, post-acute care, bundled payments, or risk adjustment;

guideline or real-world management evidence explaining variation between recommended care and actual utilization;

methodological relevance to claims-based economic interpretation, including cost attribution, confounding, exposure definition, and downstream-event accounting.

Primary KOA evidence was preferred. Knee/hip-OA or OA-wide studies were used only when they addressed mechanisms relevant to KOA interpretation — such as opioid use, NSAID contraindications, pain-related utilization, societal cost, or pharmacologic cost-effectiveness — and in those cases the population label is stated explicitly in the text.

5. Source Boundaries and Non-Prioritized Evidence

Sources were not prioritized for main-text synthesis when they lacked economic, utilization, pharmacologic, or pathway relevance. Purely technical surgical studies without extractable economic or resource-use data were not emphasized. Clinical efficacy studies were included only when they

directly informed economic interpretation, such as corticosteroid safety, PRP utility assumptions, or injection-related downstream-event uncertainty.

Non-human studies, conference abstracts without sufficient data, non-knee-OA studies without relevant knee-specific or pathway-relevant implications, and sources without interpretable cost, utilization, exposure, or decision-relevant outcomes were not used as core evidence. Prior systematic reviews and meta-analyses were used to contextualize major evidence families and identify interpretive patterns, but primary claims, registry, cohort, trial-based, and model-based studies were preferred for numerical statements whenever available.

English-language publications, or publications with sufficient English-language information for reliable extraction, were prioritized for core synthesis. No geographic restrictions were applied; however, non-English full texts without sufficient extractable English-language data were not retained for the main narrative synthesis.

6. Source-Charting Domains

For sources discussed in the main synthesis, decision-relevant information was charted when available. Charted elements included: country or health-system context; data source and study design; KOA, knee/hip-OA, or OA-wide population definition; sample size; intervention or exposure definition; comparator; pathway stage; follow-up duration or time horizon; analytic perspective; cost year and currency; costing basis (paid claims, allowed amounts, reimbursement, tariffs, charges, or modeled costs); utilization outcomes; drug or injection exposure measures; costs, QALYs, ICERs, net monetary benefit, or price-threshold results; sensitivity analyses; funding source and declared conflicts of interest, when reported; and major limitations affecting interpretation.

Cost values were retained in the currency and price year reported by each source unless the cited study itself had standardized values. This decision was made to avoid introducing misleading cross-country comparability. When multiple countries or payer systems were discussed, the synthesis emphasized relative interpretation rather than pooled cost estimates. The charted domains correspond to the columns of Supplementary Table S1, which records this information for the retained core evidence sources.
