# Supplementary material for: Beyond the Surgical Bill: Pharmacoeconomics and Real-World Utilization Across the Knee Osteoarthritis Care Pathway—A Critical Narrative Review
Source: Healthcare (Basel). 2026 Jul 9;14(14):2066. doi: 10.3390/healthcare14142066 (PMC13409983; doi:10.3390/healthcare14142066)
Supplement: Supplementary file 1 [file healthcare-14-02066-s001.zip › Supplementary Table S2.pdf]

Supplementary Table S2 — Source-identification summary

Descriptive source-identification table

Supplementary Table S2. Descriptive source-identification summary for the critical narrative review. Counts summarize how candidate sources were narrowed to the core evidence base used in the synthesis.

Source-identification step	Records, n
Candidate records identified (PubMed, Web of Science, Scopus, Google Scholar + citation tracking)	571
Duplicate records removed	173
Records reviewed for title/abstract relevance	398
Reports sought for full-text retrieval	98
Full-text reports reviewed for narrative scope/fit	88
**Core evidence sources summarized in Supplementary Table S1**	**83**

Five full-text reports were not prioritized as core evidence because they lacked direct economic, utilization, pharmacologic, or pathway relevance; duplicated evidence already captured by more direct primary sources; or did not provide extractable cost, exposure, or decision-relevant information.

Search window: publications 1 January 2000 – 30 April 2026; searches run 1–30 April 2026. The synthesis is based on 83 core evidence sources, summarized in Supplementary Table S1; additional references are cited only for contextual purposes and are not counted as core evidence sources.
